# Association of Axillary Dissection With Systemic Therapy in Patients With Clinically Node-Positive Breast Cancer

**DOI:** 10.1001/jamasurg.2023.2840

**Published:** 2023-07-19

**Authors:** Walter P. Weber, Zoltan Matrai, Stefanie Hayoz, Christoph Tausch, Guido Henke, Frank Zimmermann, Giacomo Montagna, Florian Fitzal, Michael Gnant, Thomas Ruhstaller, Simone Muenst, Andreas Mueller, Loïc Lelièvre, Jörg Heil, Michael Knauer, Daniel Egle, Ákos Sávolt, Martin Heidinger, Christian Kurzeder, and the TAXIS Study Writing Group, Daniel R. Zwahlen, Günther Gruber, Markus Ackerknecht, Sherko Kuemmel, Vesna Bjelic-Radisic, Viktor Smanykó, Conny Vrieling, Rok Satler, Daniela Hagen, Charles Becciolini, Susanne Bucher, Colin Simonson, Peter M. Fehr, Natalie Gabriel, Robert Maráz, Dimitri Sarlos, Konstantin J. Dedes, Cornelia Leo, Gilles Berclaz, Hisham Fansa, Christopher Hager, Klaus Reisenberger, Christian F. Singer, Sibylle Loibl, Jelena Winkler, Giang Thanh Lam, Mathias K. Fehr, Magdalena Kohlik, Karine Clerc, Valerijus Ostapenko, Nadia Maggi, Alexandra Schulz, Mariacarla Andreozzi, Maite Goldschmidt, Ramon Saccilotto, Pagona Markellou

**Affiliations:** 1 Breast Center, University Hospital Basel, Basel, Switzerland; 2 Faculty of Medicine, University of Basel, Basel, Switzerland; 3 Hamad Medical Corporation, Dept of Oncoplastic Breast Surgery, Doha, Qatar; 4 Competence Center of SAKK, Bern, Switzerland; 5 Breast Center Zurich, Zurich, Switzerland; 6 Department of Radiation Oncology, St Gallen Cantonal Hospital, St Gallen, Switzerland; 7 Breast Center, St Gallen Cantonal Hospital, St Gallen, Switzerland; 8 Clinic of Radiation Oncology, University Hospital Basel, Basel, Switzerland; 9 Breast Service, Department of Surgery, Memorial Sloan Kettering Cancer Center, New York, New York; 10 Department of Surgery, Medical University Vienna, Vienna, Austria; 11 Comprehensive Cancer Center Medical University Vienna, Vienna, Austria; 12 Austrian Breast and Colorectal Cancer Study Group, Vienna, Austria; 13 Tumor and Breast Center Eastern Switzerland, St Gallen, Switzerland; 14 Institute of Medical Genetics and Pathology, University Hospital Basel, Basel, Switzerland; 15 Breast Center, Cantonal Hospital Winterthur, Winterthur, Switzerland; 16 Breast Center, Centre Hospitalier Universitaire Vaudois, Lausanne, Switzerland; 17 Breast Center Heidelberg, Heidelberg, Germany; 18 Breast Cancer Center Tirol, Department of Gynecology, Medical University Innsbruck, Innsbruck, Austria; 19 Department of Breast and Sarcoma Surgery, National Institute of Oncology, Budapest, Hungary; 20 Department of Radiation Oncology, Cantonal Hospital Winterthur, Winterthur, Switzerland; 21 Institute of Radiotherapy, Klinik Hirslanden, Zurich, Switzerland; 22 Department of Biomedicine, University Hospital Basel, Basel, Switzerland; 23 Breast Unit, Kliniken Essen-Mitte, Essen, Charité, Germany; 24 Department of Gynecology with Breast Center, Universitätsmedizin Berlin, Berlin, Germany; 25 Breast Unit, Helios University Clinic, University Witten/Herdecke, Germany; 26 Centre of Radiotherapy, National Institute of Oncology, Budapest, Hungary; 27 Department of Radiation Oncology, Hirslanden Clinique des Grangettes, Geneva, Switzerland; 28 Breast Center, Réseau Hospitalier Neuchâtelois, La Chaux-de-Fonds, Switzerland; 29 Breast Center, Cantonal Hospital Lucerne, Lucerne, Switzerland; 30 Department of Gynecology, Centre Hospitalier du Valais Romand, Hôpital de Sion, Switzerland; 31 Breast Center Graubünden, Cantonal Hospital Graubünden, Chur, Switzerland; 32 Breast Center, City Hospital Zurich Triemli, Zurich, Switzerland; 33 Department of Oncology, Bacs-Kiskun Country Hospital, Kecskemet, Hungary; 34 Breast Center, Cantonal Hospital Aarau, Aarau, Switzerland; 35 Breast Cancer Center, Zurich Lake, Zurich, Switzerland; 36 Breast Center, Cantonal Hospital Baden, Baden, Switzerland; 37 Breast Center Bern, Lindenhof group, Bern, Switzerland; 38 Breast Center Zürich, Bethanien & Spital Zollikerberg, Zurich, Switzerland; 39 Department of Gynecology and Obstetrics, City Hospital, Dornbirn, Austria; 40 Department of Gynecology and Obstetrics, Klinikum Wels-Grieskirchen, Wels, Austria; 41 Department of Gynecology and Obstetrics and Comprehensive Cancer Center, Medical University of Vienna, Vienna, Austria; 42 German Breast Group, GBG Forschungs-GmbH, Neu-Isenburg, Germany; 43 Breast Center, Basel Bethesda Hospital, Basel, Switzerland; 44 Breast Center, University Hospital of Geneva, Geneva, Switzerland; 45 Breast Center Thurgau, Frauenfeld, Switzerland; 46 Centre du Sein GSMN, Clinique de Genolier, Genolier, Switzerland; 47 Brustzentrum Freiburg, Centre du sein Fribourg, Fribourg, Switzerland; 48 National Cancer Institute, Vilnius, Lithuania; 49 Department of Clinical Research, University Hospital Basel, Basel, Switzerland

## Abstract

**Importance:**

The role of axillary lymph node dissection (ALND) to determine nodal burden to inform systemic therapy recommendations in patients with clinically node (cN)–positive breast cancer (BC) is currently unknown.

**Objective:**

To address the association of ALND with systemic therapy in cN-positive BC in the upfront surgery setting and after neoadjuvant chemotherapy (NACT).

**Design, Setting, and Participants:**

This was a prospective, observational, cohort study conducted from August 2018 to June 2022. This was a preplanned study within the phase 3 randomized clinical OPBC-03/TAXIS trial. Included were patients with confirmed cN-positive BC from 44 private, public, and academic breast centers in 6 European countries. After NACT, residual nodal disease was mandatory, and a minimum follow-up of 2 months was required.

**Exposures:**

All patients underwent tailored axillary surgery (TAS) followed by ALND or axillary radiotherapy (ART) according to TAXIS randomization. TAS removed suspicious palpable and sentinel nodes, whereas imaging-guidance was optional. Systemic therapy recommendations were at the discretion of the local investigators.

**Results:**

A total of 500 patients (median [IQR] age, 57 [48-69] years; 487 female [97.4%]) were included in the study. In the upfront surgery setting, 296 of 335 patients (88.4%) had hormone receptor (HR)–positive and Erb-B2 receptor tyrosine kinase 2 (*ERBB2*; formerly *HER2* or *HER2/neu*)–negative disease: 145 (49.0%) underwent ART, and 151 (51.0%) underwent ALND. The median (IQR) number of removed positive lymph nodes without ALND was 3 (1-4) nodes compared with 4 (2-9) nodes with ALND. There was no association of ALND with the proportion of patients undergoing adjuvant chemotherapy (81 of 145 [55.9%] vs 91 of 151 [60.3%]; adjusted odds ratio [aOR], 0.72; 95% CI, 0.19-2.67) and type of systemic therapy. Of 151 patients with NACT, 74 (51.0%) underwent ART, and 77 (49.0%) underwent ALND. The ratio of removed to positive nodes was a median (IQR) of 4 (3-7) nodes to 2 (1-3) nodes and 15 (12-19) nodes to 2 (1-5) nodes in the ART and ALND groups, respectively. There was no observed association of ALND with the proportion of patients undergoing postneoadjuvant systemic therapy (57 of 74 [77.0%] vs 55 of 77 [71.4%]; aOR, 0.86; 95% CI, 0.43-1.70), type of postneoadjuvant chemotherapy (eg, capecitabine: 10 of 74 [13.5%] vs 10 of 77 [13.0%]; trastuzumab emtansine–DM1: 9 of 74 [12.2%] vs 11 of 77 [14.3%]), or endocrine therapy (eg, aromatase inhibitors: 41 of 74 [55.4%] vs 36 of 77 [46.8%]; tamoxifen: 8 of 74 [10.8%] vs 6 of 77 [7.8%]).

**Conclusion:**

Results of this cohort study suggest that patients without ALND were significantly understaged. However, ALND did not inform systemic therapy recommendations.

## Key Points

**Question:**

Is the omission of axillary dissection in patients with clinically node-positive breast cancer associated with systemic therapy in the upfront surgery and postneoadjuvant setting?

**Findings:**

In this international cohort study embedded in a randomized clinical trial, a total of 500 patients had a high nodal tumor burden and were significantly understaged when axillary dissection was omitted. Nevertheless, this did not change adjuvant and postneoadjuvant systemic therapy.

**Meaning:**

Results of this cohort study suggest that axillary dissection did not inform systemic therapy recommendations in individuals with clinically node-positive breast cancer.

## Teaser

This cohort study investigates the association between the omission of axillary dissection and systemic therapy in patients with clinically node-positive breast cancer in the postneoadjuvant setting.

## Introduction

Standard axillary lymph node dissection (ALND) was replaced by the sentinel lymph node (SLN) procedure in most patients with node-negative breast cancer (BC).^1,2^ In patients with clinically node-negative SLN-positive BC, several landmark trials showed noninferior survival and recurrence when ALND was omitted.^3,4,5^ Limited knowledge of the exact number of positive nodes did not modify the likelihood to receive chemotherapy in these trials.^3,5^ The staging role of ALND has been further diminished because chemotherapy is increasingly based on tumor biology, which currently applies to most patients with node-positive triple-negative (TN) or Erb-B2 receptor tyrosine kinase 2 (*ERBB2*; formerly *HER2* or *HER2/neu*)–positive BC.^6,7^

However, in patients with hormone receptor (HR)–positive *ERBB2*-negative BC, the indication for chemotherapy may still depend on the total number of positive nodes. Traditionally, most experts recommended chemotherapy in patients with luminal BC and 4 or more positive nodes.^7^ More recently, genomic assays, such as the Oncotype DX Recurrence Score (Exact Sciences) or Mammaprint (Agendia), became available to refine chemotherapy indications in node-positive, HR-positive, *ERBB2*-negative BC.^8,9^ Because patients with more than 3 positive nodes were ineligible for these trials, applicability of their results to patients who did not undergo ALND remains questionable. Similarly, the recent MONARCHE trial raised the question of whether the exact number of positive nodes is required to indicate the cyclin-dependent kinase 4/6 (CDK4/6) inhibitor, abemaciclib, after upfront surgery.^10^ Finally, in the postneoadjuvant setting, response-driven therapy is increasingly used and may be influenced by surgical staging of the axilla.^11,12^

To our knowledge, the role of ALND to determine the nodal tumor burden to inform systemic therapy decisions in patients with clinically node (cN)–positive BC is not known. Oncoplastic Breast Consortium 03 (OPBC-03)/Tailored Axillary Surgery With or Without Axillary Lymph Node Dissection Followed by Radiotherapy in Patients With Clinically Node-positive Breast Cancer (TAXIS) is an ongoing, international, phase 3 surgical trial investigating noninferiority (with regard to disease-free survival [DFS], but with strong emphasis on quality of life) of axillary radiotherapy (ART) relative to ALND in the treatment of patients with cN-positive BC who undergo tailored axillary surgery (TAS).^13^ Publication of the first prespecified substudy showed that patients in the ART arm were significantly understaged, with 70% of patients in the ALND arm having further nodal disease removed by ALND, of whom 37% had 4 or more additional positive nodes.^14^ The present study was planned to gain relevant insight on the association of TAS with the use of adjuvant and postneoadjuvant systemic treatment, which, in turn, may be associated with the primary end point of the main trial. The aim of this analysis was to assess the role of ALND as a decision aid for systemic therapy in a contemporary cohort of patients with cN-positive BC in the upfront surgery and postneoadjuvant setting.

## Methods

This study represents a prospective, observational, cohort study within the pragmatic, phase 3, international, multicenter, randomized clinical TAXIS trial.^13^ The trial was approved by the local ethics committees and was performed in accordance with the requirements of the national regulatory authorities. Written informed consent was obtained from all patients. Included were patients with cN-positive BC, defined as nodal disease detected by palpation or imaging at the time of initial diagnosis and histologic or cytologic confirmation both in the primary tumor and lymph node metastasis. According to the pragmatic design, patients were included in the upfront surgery and neoadjuvant setting, while in the latter, confirmation of residual nodal disease at the time of surgery was mandatory. Patients with stage IV, cN3c, or cN2b BC; contralateral or other tumor malignancy within 3 years; and prior axillary surgery (except SLN biopsy) or prior axillary radiotherapy were excluded. Systemic therapy recommendations were at the discretion of the local investigators. All drugs used for adjuvant systemic anticancer treatment as chosen based on international and/or local guidelines were recorded. Follow-up was predefined in the study protocol.^13^ The sample size of the main TAXIS trial was 1500 patients (750 per arm) and was based on the primary end point DFS. The patient population in the present study was a priori defined to include patients randomly assigned to treatment groups who were treated from August 2018 to June 2022 to address the association of staging information gleaned from ALND with adjuvant systemic therapy treatment decisions. The 2 groups of patients were as follows: (1) patients with cN-positive BC who underwent upfront surgery and (2) patients with cN-positive BC with persistent nodal involvement after neoadjuvant chemotherapy (NACT). Minimum follow-up after surgery was 2 months. Data extraction was performed after data cleaning on September 30, 2022. No participants were lost to follow-up until this analysis was performed. Participant race and ethnicity data were not gathered for this study as it was not planned in the study protocol. This study followed the Strengthening the Reporting of Observational Studies in Epidemiology (STROBE) reporting guidelines.^15^

### Locoregional Treatment

The initially sampled and histologically or cytologically positive node was marked with a clip. TAS was defined by removal of SLNs (detection modality choice left up to surgeon; dual tracer not required) and palpable lymph node metastases; localization of the previously clipped biopsied node (which is a standard part of targeted axillary dissection) is not mandatory.^14^ Importantly, in the experimental setting of TAS, palpation-guided removal of suspicious nodes is a key component, whereas palpable disease is usually a contraindication to the SLN procedure in today’s clinical practice. Intraoperative exclusion criteria for surgical quality control included failure to remove (1) the clip, (2) at least 1 SLN, and (3) all suspicious palpable findings. Patients in the control group of the TAXIS trial underwent TAS followed by ALND after intraoperative randomization. When a patient was randomly assigned to the ALND group, the procedure was technically performed according to the standard of the treating surgeon in line with the pragmatic study design. However, the protocol required levels I and II to be cleared and a full level III dissection above and medial to the pectoralis minor muscle to be carried out only when there was gross nodal disease detected by palpation or imaging. In the other arm of the TAXIS trial, patients received ART. All patients underwent adjuvant whole-breast irradiation after breast-conserving surgery and chest wall irradiation after mastectomy. Although patients in the ALND group received regional nodal irradiation excluding the dissected axilla as a target volume, patients in the ART group received regional nodal irradiation including the axilla.

### End Points

The primary end point for this substudy was patients undergoing adjuvant chemotherapy.^13^ Secondary end points included patients undergoing postneoadjuvant systemic therapy, type of chemotherapy, and type of endocrine therapy. All of these end points were prespecified. Post hoc analyses included comparisons of differences in systemic therapies other than chemotherapy and endocrine therapy.

### Statistical Analysis

Continuous end points were summarized using median and IQR, and the Hodges-Lehmann estimator with corresponding 95% CI was used to report differences between treatment arms. Categorical end points were summarized using frequency counts and percentages as well as differences in proportions between treatment arms with corresponding 95% CI. Due to the exploratory nature of this analysis, no hypothesis testing was performed. To assess the association of treatment arm with the administration of adjuvant systemic therapy and chemotherapy, unadjusted odds ratio (OR) as well as adjusted OR (aOR) with corresponding 95% CI for palpable vs nonpalpable disease, menopausal status, tumor subtype, tumor grade, age, year, and country were calculated using logistic regression models. All analyses were performed using R, version 4.2.1 (R Project for Statistical Computing).

## Results

A total of 500 patients (median [IQR] age, 57 [48-69] years; 487 female [97.4%]; 13 male [2.6%]) were included at 44 breast centers from 6 European countries (Table 1). Overall, included subtypes were HR positive/*ERBB2* negative in 397 patients (79.4%), HR positive/*ERBB2* positive in 52 patients (10.4%), HR negative/*ERBB2* positive in 5 patients (1.0%), and HR negative/*ERBB2* negative in 35 patients (7.0%).

**Table 1.** Clinicopathologic Characteristics of Study Cohort

Characteristic	Patients (N = 500)
Age, median (IQR), y	57 (48-69)
Sex, No. (%)	
Female	487 (97.4)
Male	13 (2.6)
Country, No. (%)	
Austria	29 (5.8)
Germany	31 (6.2)
Hungary	99 (19.8)
Italy	2 (0.4)
Lithuania	4 (0.8)
Switzerland	335 (67.0)
Menopausal status, No. (%)	
Postmenopausal	342 (68.4)
Premenopausal	157 (31.4)
Unknown	1 (0.2)
Histological findings, No. (%)	
Ductal	389 (77.8)
Lobular	60 (12.0)
Other	50 (10.0)
Unknown	1 (0.2)
Differentiation, No. (%)	
Well	32 (6.4)
Moderate	294 (58.8)
Poor	169 (33.8)
Unknown	5 (1.0)
Receptor status, No. (%)	
HR−/*ERBB2*−	35 (7.0)
HR−/*ERBB2*+	5 (1.0)
HR+/*ERBB2*−	397 (79.4)
HR+/*ERBB2*+	52 (10.4)
Unknown	11 (2.2)
Tumor size, median (IQR), mm	28 (20-40)
Unknown	17
Breast surgery, No. (%)	
Breast-conserving surgery	293 (58.6)
Mastectomy	207 (41.4)
Treatment arm, No. (%)	
Arm A: ALND	250 (50.0)
Arm B: no ALND	250 (50.0)
No. of LNs retrieved during TAS, median (IQR)	5 (3-8)
Unknown, No.	7
No. of additional LNs retrieved during ALND, median (IQR)	12 (9-17)
Unknown, No.	8
Type of clinical node positivity, No. (%)	
Nonpalpable, detected by imaging	242 (48.4)
Palpable	258 (51.6)

Abbreviations: ALND, axillary lymph node dissection; *ERBB2*−, Erb-B2 receptor tyrosine kinase 2 negative (formerly *HER2* or *HER2/neu*); *ERBB2*+, Erb-B2 receptor tyrosine kinase 2 positive; HR−, hormone receptor negative; HR+, hormone receptor positive; TAS, tailored axillary surgery.

Of 335 patients (67.0%) who were treated in the upfront surgery setting, 296 (88.4%) had HR-positive/*ERBB2*-negative disease. Of these 296 patients, 145 (49.0%) underwent ART without ALND, and 151 (51.0%) underwent ALND after TAS. In the ART arm, the median (IQR) number of lymph nodes removed was 5 (4-8) nodes, of which 3 (1-4) nodes were positive. In the ALND arm, the median (IQR) number of lymph nodes was 19 (14-26) nodes, of which 4 (2-9) nodes were positive (Table 2). Four or more positive nodes were found in 49 of 145 patients (33.8%) in the ART arm and in 89 of 151 patients (58.9%) in the ALND arm. We observed no association of ALND with the proportion of patients with HR-positive/*ERBB2*-negative disease undergoing adjuvant chemotherapy (81 of 145 [55.9%] in the ART arm and 91 of 151 [60.3%] in the ALND arm; aOR, 0.72; 95% CI, 0.19-2.67) (Table 3 and eTable in Supplement 1). Furthermore, we observed no differences in type of systemic therapy with the exception of tamoxifen, which was 30 of 151 (19.9%) with ALND vs 13 of 145 (9.0%) without ALND (Table 4).

**Table 2.** Number of Lymph Nodes Removed by Type of Surgery

No. of lymph nodes removed	Type of surgery		Difference (95% CI)^a^
	Arm A: ALND (n = 250)	Arm B: no ALND (n = 250)	
Overall, median (IQR)			
Total	18 (13-24)	5 (3-7)	12 (11-13)
Positive	4 (2-8)	2 (1-4)	1 (1-2)
Negative	12 (8-16)	2 (1-4)	9 (8-10)
Upfront surgery setting: HR+/*ERBB2*−, median (IQR)			
Total No.	151	145	NA
Total	19 (14-26)	5 (4-8)	14 (12-15)
Positive	4 (2-9)	3 (1-4)	2 (1-2)
Negative	12 (9-18)	2 (1-4)	10 (9-11)
Neoadjuvant chemotherapy,^b^ median (IQR)			
Total No.	77	74	NA
Total	15 (12-19)	4 (3-7)	10 (9-12)
Positive	2 (1-5)	2 (1-3)	1 (0-1)
Negative	12 (7-15)	2 (1-4)	9 (7-11)

Abbreviations: ALND, axillary lymph node dissection; *ERBB2*−, Erb-B2 receptor tyrosine kinase 2 negative (formerly *HER2* or *HER2/neu*); HR+, hormone receptor positive; NA, not applicable.

^a^ Hodges-Lehmann estimator.

^b^ Fourteen patients had other neoadjuvant therapy than chemotherapy.

**Table 3.** Proportion of Patients Undergoing Chemotherapy With or Without Axillary Lymph Node Dissection (ALND)

Group	Arm A: ALND	Arm B: No ALND	Difference (95% CI)^a^
Upfront surgery setting, No./total No. (%)			
All subtypes	101/169 (59.8)	92/166 (55.4)	4.3% (−6.8% to 16%)
HR+/*ERBB2*−	91/151 (60.3)	81/145 (55.9)	4.4% (−7.5% to 16%)
HR+/*ERBB2*− Premenopausal	36/47 (76.6)	28/37 (75.7)	0.9% (−18% to 20%)
HR+/*ERBB2*− Postmenopausal	55/104 (52.9)	53/108 (49.1)	3.8% (−11% to 18%)
Neoadjuvant chemotherapy,^b^ No./total No. (%)			
All subtypes	24/77 (31.2)	20/74 (27.0)	4.1% (−12% to 20%)
HR+/*ERBB2*−	5/41 (12.2)	8/48 (16.7)	−4.5% (−21% to 12%)
HR+/*ERBB2*+	11/20 (55.0)	7/12 (58.3)	−3.3% (−42% to 35%)
HR−/*ERBB2*−	7/14 (50.0)	4/12 (33.3)	17% (−28% to 62%)
HR−/*ERBB2*+	0/1 (0)	1/1 (100)	−100% (−100% to 0%)
Premenopausal	11/27 (40.7)	10/34 (29.4)	11% (−16% to 39%)
Postmenopausal	13/50 (26.0)	10/40 (25.0)	1% (−18% to 20%)

Abbreviations: *ERBB2*−, Erb-B2 receptor tyrosine kinase 2 negative (formerly *HER2* or *HER2/neu*); *ERBB2*+, Erb-B2 receptor tyrosine kinase 2 positive; HR−, hormone receptor negative; HR+, hormone receptor positive.

^a^ Difference in proportions.

^b^ Fourteen patients had other neoadjuvant therapy than chemotherapy.

**Table 4.** Type of Adjuvant Systemic Therapy in Patients With Hormone Receptor–Positive and Erb-B2 Receptor Tyrosine Kinase 2 (*ERBB2*)^a^–Negative Subtype by Type of Surgery in the Upfront Surgery Setting

Type of therapy	No. (%)		Difference (95% CI)^b^
	Arm A: ALND (n = 151)	Arm B: no ALND (n = 145)	
Taxane-containing chemotherapy	80 (53.0)	72 (49.7)	3.3% (−8.7% to 15%)
Anthracycline-containing chemotherapy	78 (51.7)	64 (44.1)	7.5 (−4.5% to 20%)
Nontaxane/nonanthracycline–containing chemotherapy	88 (58.3)	76 (52.4)	5.9% (−6.1% to 18%)
Carboplatin/cisplatin	0 (0)	3 (2.1)	−2.1% (−5.1% to 0.92%)
Aromatase inhibitors	90 (59.6)	89 (61.4)	−1.8% (−14% to 10%)
GnRH analogs	17 (11.3)	10 (6.9)	4.4% (−2.8% to 12%)
Fulvestrant	1 (0.7)	2 (1.4)	−0.7 (−3.7% to 2.3%)
Tamoxifen	30 (19.9)	13 (9.0)	11% (2.3% to 19%)
CDK4/6 inhibitors	1 (0.7)	3 (2.1)	−1.4 (−4.7% to 1.9%)

Abbreviations: ALND, axillary lymph node dissection; CDK4/6, cyclin-dependent kinase 4/6; GnRH, gonadotropin-releasing hormone.

^a^ Formerly *HER2* or *HER2/neu*.

^b^ Difference in proportions.

A total of 151 of 500 patients (30.2%) underwent NACT, 13 had neoadjuvant endocrine treatment, and 1 had neoadjuvant double *ERBB2* blockade without chemotherapy. Of the 151 patients who received NACT with residual nodal disease, 74 (49.0%) underwent ART without ALND and 77 (51.0%) underwent ALND. In the ART arm, the median (IQR) number of lymph nodes removed was 4 (3-7) nodes, of which 2 (1-3) nodes were positive; in the ALND arm, the number was 15 (12-19) nodes, of which 2 (1-5) nodes were positive (Table 2). We observed no association of ALND in patients after neoadjuvant treatment with the proportion of patients undergoing postneoadjuvant systemic therapy (57 of 74 [77.0%] in the ART arm and 55 of 77 [71.4%] in the ALND arm; aOR, 0.86; 95% CI, 0.43-1.70) (Table 3 and eTable in Supplement 1). Furthermore, no differences in type of postneoadjuvant chemotherapy (eg, capecitabine: 10 of 74 [13.5%] vs 10 of 77 [13.0%]; trastuzumab emtansine–DM1: 9 of 74 [12.2%] vs 11 of 77 [14.3%]) or endocrine therapy (eg, aromatase inhibitors: 41 of 74 [55.4%] vs 36 of 77 [46.8%]; tamoxifen: 8 of 74 [10.8%] vs 6 of 77 [7.8%]) were observed (Table 5).

**Table 5.** Type of Postneoadjuvant Systemic Therapy by Type of Surgery

Type of therapy	No. (%)		Difference (95% CI)^a^
	Arm A: ALND (n = 77)^a^	Arm B: No ALND (n = 74)^a^	
Carboplatin/cisplatin	3 (3.9)	0 (0)	3.9% (−1.8% to 9.5%)
Capecitabine	10 (13.0)	10 (13.5)	−0.5% (−12% to 11%)
Trastuzumab	5 (6.5)	5 (6.8)	−0.3% (−8.5% to 7.9%)
Pertuzumab	1 (1.3)	1 (1.4)	−0.1% (−3.8% to 3.6%)
T-DM1	11 (14.3)	9 (12.2)	2.1% (−10% to 14%)
Checkpoint inhibitors	2 (2.6)	0 (0)	2.6% (−2.3% to 7.5%)
Aromatase inhibitors	36 (46.8)	41 (55.4)	−8.7% (−26% to 8.6%)
GnRH analogons	4 (5.2)	7 (9.5)	−4.3% (−14% to 5.4%)
Tamoxifen	6 (7.8)	8 (10.8)	−3.0% (−14% to 7.6%)
CDK4/6 inhibitors	3 (3.9)	4 (5.4)	−1.5% (−9.6% to 6.5%)
PARP inhibitors	0 (0)	1 (1.4)	−1.4% (−5.3% to 2.6%)

Abbreviations: ALND, axillary lymph node dissection; CDK4/6, cyclin-dependent kinase 4/6; GnRH, gonadotropin-releasing hormone; PARP, poly ADP ribose polymerase; T-DM1, trastuzumab emtansine–DM1.

^a^ Difference in proportions.

## Discussion

To the authors’ knowledge, this was the first study to prospectively assess the role of ALND to determine nodal tumor burden to inform systemic therapy in patients with cN-positive BC. The main finding was that use of ALND and associated detailed knowledge of the number of positive nodes did not significantly change systemic therapy in the upfront surgery and postneoadjuvant setting. All patients underwent TAS, which was shown to selectively remove positive nodes while remaining much less radical than standard ALND.^14^ The vast majority of patients had luminal cancers. Because other subtypes were less commonly treated in the adjuvant setting and in the neoadjuvant setting, patients with these subtypes had a higher likelihood of being excluded due to nodal pathologic complete response (pCR). In fact, the present findings are of particular interest in patients with HR-positive/*ERBB2*-negative cN-positive BC, with 91 of 151 patients (60%) undergoing adjuvant chemotherapy when ALND is used, compared with 81 of 145 patients (56%) without ALND. The total number of 4 positive nodes removed in the ALND group reflects the traditional threshold for chemotherapy in luminal breast cancer, whereas only a median of 3 nodes were removed without ALND.^7^ In fact, the proportion of patients with 4 or more positive lymph nodes was 89 of 151 (59%) and 49 of 145 (34%) with vs without ALND, respectively. This is substantially higher than in the American College of Surgeons Oncology Group Z0011 trial, where the corresponding numbers were 14% vs 1%.^3^ Therefore, the present results suggest that even in a patient population with high nodal burden that is likely to be heavily understaged when ALND is omitted, the use of ALND to determine the exact number of positive nodes does not change systemic therapy. The only observed difference was the higher use of tamoxifen after ALND in ER-positive/*ERBB2*-negative BC. Because age and menopausal status were well balanced and the number of positive nodes was high with no difference between the groups, we could not determine why clinicians felt confident to use tamoxifen over an aromatase inhibitor in the ALND group. However, these associations will be reevaluated once the full TAXIS sample size is reached.

In recent years, gene expression profiles have been introduced to provide additional prognostic information in conjunction with nodal stage to inform systemic therapy recommendations in patients with luminal BC.^8,9^ In the Clinical Trial Rx for Positive-Node, Endocrine-Responsive Breast Cancer (RxPONDER) trial, women with HR-positive, *ERBB2*-negative BC, 1 to 3 positive axillary lymph nodes, and an Oncotype DX Recurrence Score of 25 or lower were randomly assigned to endocrine therapy only or to chemotherapy plus endocrine therapy.^9^ At 5 years, the invasive DFS rate among postmenopausal women was not different in the endocrine therapy–only group (91.9%) vs the chemoendocrine therapy group (91.3%). Based on these results, chemotherapy is not indicated in postmenopausal women selected accordingly. Importantly, in the RxPONDER trial, only 62% of patients were staged by ALND, and 37% underwent SLN biopsy alone with presumed nodal understaging. The second landmark trial that introduced molecular assays to refine chemotherapy indications in node-positive BC was the Microarray in Node-Negative and 1 to 3 Positive Lymph Node Disease May Avoid Chemotherapy (MINDACT) trial.^8^ In the initial study design, all patients had to have lymph node–negative disease, but about halfway through, the protocol was revised to allow for the enrollment of women with up to 3 positive axillary nodes. Because both trials did not allow inclusion of patients with more than 3 positive nodes, to determine the total tumor load in the axilla and assess eligibility for these tests to inform systemic therapy, ALND was revisited.^16^ Although the main results of the RxPONDER trial were published rather recently, those of the MINDACT trial came out before this study started, and we did not observe any time trend during the study period (data not shown) or differences by menopausal status (Table 3). Therefore, the present results suggest that clinicians were not considering the total number of positive nodes as a major factor in the decision for use of chemotherapy, either directly or through use of molecular tests.

The present study cannot address the impact of the MONARCHE trial on use of ALND for staging purposes in patients with cN-positive BC because it was published toward the end of the study period.^10^ Nodal stage was used for risk stratification, and patients with high-risk early-stage HR-positive disease were randomly assigned to receive endocrine therapy with the CDK4/6 inhibitor abemaciclib or endocrine therapy alone in the adjuvant setting. A significant improvement in invasive DFS could be demonstrated for high-risk patients defined as having either 4 or more positive nodes or 1 to 4 positive nodes with additional risk factors. Given the importance of the extent of nodal disease in MONARCHE, surgical management of the axilla has resurfaced as a question asked at multidisciplinary tumor boards. ALND is sometimes recommended by medical oncologists to assess eligibility for application of the trial protocol to individual patients in clinical practice.^16^ In the present study, only 4 patients received CDK4/6 inhibitors, which may be explained by the initially conflicting results and the still hesitant adoption of the use of adjuvant CDK4/6 inhibition by the community, and the fact that the only positive trial, MONARCHE, was just recently published with short follow-up.^10,17^

The present study will be repeated once all 1500 patients have been randomly assigned to treatment groups in the TAXIS trial to investigate if future study findings may strengthen the role of ALND to determine the total number of positive nodes. However, morbidity is well documented to be increased after ALND compared with the SLN procedure.^18,19,20,21^ Even though the SLN procedure was primarily introduced in clinical practice to stage the node-negative axilla with less harm for patients, several landmark trials established the safety of ALND omission in patients with cN-negative BC and positive SLN.^3,4,5,22,23,24,25^ Ongoing trials such as the present OPBC-03/TAXIS and Alliance for Clinical Trials in Oncology A011202 aim at further de-escalating surgical treatment of the axilla by showing noninferiority of ART compared with ALND even in cN-positive BC.^13,26^ Therefore, escalating axillary surgery for staging purposes seems counterintuitive, and we anticipate that surgeons are reluctant to go back and perform ALND for this reason, which is in line with the main findings of this study.

In the postneoadjuvant setting, patients with residual triple-negative disease showed improved oncologic outcomes when receiving capecitabine as did patients with residual *ERBB2*-positive disease when receiving trastuzumab emtansine–DM1.^11,12^ In both trials, patients with tumor-positive lymph nodes were also eligible. Therefore, type of axillary surgery has the potential to influence response-driven chemotherapy in case of pCR in the breast and residual nodal disease that is missed by lesser surgical staging of the axilla. There are 2 reasons why this scenario is unlikely. First, the false-negative rate of TAS was shown to be as low as 2.6%.^14^ Second, breast pCR is a strong predictor of nodal pCR. Data from an exploratory analysis within the Randomized Phase 3 Trial Comparing 2 Dose-Dense, Dose-Intensified Approaches (ETC and PM[Cb]) for Neoadjuvant Treatment of Patients With High-Risk Early Breast Cancer (GeparOcto) trial showed a breast pCR rate of 45.0%, of which 91.7% also showed axillary pCR.^27^ This association was confirmed by a Korean trial as well as a Canadian series, showing axillary pCR in breast pCR in 86.6% and 83.0% of patients, respectively.^28,29^ Because in the present study all included patients had confirmed residual disease in the nodes, the lack of differences in use of postneoadjuvant systemic therapy by type of axillary surgery was to be expected.

### Limitations

This was a prospective observational cohort study embedded in a randomized clinical trial. Accordingly, although the 2 groups with or without ALND were well balanced for patient, tumor, and treatment characteristics as well as other known prognostic variables, residual confounding by unknown factors cannot be excluded. In addition, the present study was primarily designed to investigate the association of ALND use with proportion and type of systemic therapy; other aspects including, eg, density of dosing, were not addressed. Dose-dense adjuvant chemotherapy has been shown to improve DFS compared with standard interval chemotherapy in patients with node-positive early BC.^30^ In patients who had 4 or more positive axillary lymph nodes, dose intensification has been shown to significantly improve event-free survival.^31^ It would have been interesting to see if ALND had an impact on dose density.

## Conclusions

Results of this cohort study suggest that omission of ALND was associated with understaging in patients with cN-positive BC with a high tumor burden in the axilla. However, missing knowledge of the exact number of positive nodes did not have a relevant impact on adjuvant and postneoadjuvant systemic treatment decisions. Therefore, although ALND may be considered in individual patients being treated by a multidisciplinary team, results of the present study suggest that nodal burden as determined by TAS without ALND does not generally result in underuse of systemic therapy.

## Notes

Supplement 1. **eTable.** Logistic Regression Model for the Effect of Treatment Arm on the Administration of Adjuvant Systemic Therapy and Chemotherapy

Supplement 2. Data Sharing Statement

## References

[1] Veronesi U, Paganelli G, Viale G, . A randomized comparison of sentinel-node biopsy with routine axillary dissection in breast cancer. N Engl J Med. 2003;349(6):546-553. doi:10.1056/NEJMoa012782 12904519

[2] Krag DN, Anderson SJ, Julian TB, . Sentinel lymph node resection compared with conventional axillary lymph node dissection in clinically node-negative patients with breast cancer: overall survival findings from the NSABP B-32 randomised phase 3 trial. Lancet Oncol. 2010;11(10):927-933. doi:10.1016/S1470-2045(10)70207-2 20863759 PMC3041644

[3] Giuliano AE, McCall L, Beitsch P, . Locoregional recurrence after sentinel lymph node dissection with or without axillary dissection in patients with sentinel lymph node metastases: the American College of Surgeons Oncology Group Z0011 randomized trial. Ann Surg. 2010;252(3):426-432. doi:10.1097/SLA.0b013e3181f08f32 20739842 PMC5593421

[4] Galimberti V, Cole BF, Zurrida S, ; International Breast Cancer Study Group Trial 23-01 investigators. Axillary dissection vs no axillary dissection in patients with sentinel-node micrometastases (IBCSG 23-01): a phase 3 randomised controlled trial. Lancet Oncol. 2013;14(4):297-305. doi:10.1016/S1470-2045(13)70035-4 23491275 PMC3935346

[5] Donker M, van Tienhoven G, Straver ME, . Radiotherapy or surgery of the axilla after a positive sentinel node in breast cancer (EORTC 10981-22023 AMAROS): a randomised, multicentre, open-label, phase 3 noninferiority trial. Lancet Oncol. 2014;15(12):1303-1310. doi:10.1016/S1470-2045(14)70460-7 25439688 PMC4291166

[6] National Comprehensive Cancer Network. NCCN guidelines—breast cancer. Accessed June 11, 2023. https://www.nccn.org/guidelines/guidelines-detail?category=1&id=1419

[7] Coates AS, Winer EP, Goldhirsch A, ; Panel Members. Tailoring therapies–improving the management of early breast cancer: St Gallen International Expert Consensus on the Primary Therapy of Early Breast Cancer 2015. Ann Oncol. 2015;26(8):1533-1546. doi:10.1093/annonc/mdv221 25939896 PMC4511219

[8] Cardoso F, van’t Veer LJ, Bogaerts J, ; MINDACT Investigators. 70-Gene signature as an aid to treatment decisions in early-stage breast cancer. N Engl J Med. 2016;375(8):717-729. doi:10.1056/NEJMoa1602253 27557300

[9] Kalinsky K, Barlow WE, Gralow JR, . 21-Gene assay to inform chemotherapy benefit in node-positive breast cancer. N Engl J Med. 2021;385(25):2336-2347. doi:10.1056/NEJMoa2108873 34914339 PMC9096864

[10] Johnston SRD, Harbeck N, Hegg R, ; monarchE Committee Members and Investigators. Abemaciclib combined with endocrine therapy for the adjuvant treatment of HR+, *HER2*−, node-positive, high-risk, early breast cancer (monarchE). J Clin Oncol. 2020;38(34):3987-3998. doi:10.1200/JCO.20.02514 32954927 PMC7768339

[11] Masuda N, Lee SJ, Ohtani S, . Adjuvant capecitabine for breast cancer after preoperative chemotherapy. N Engl J Med. 2017;376(22):2147-2159. doi:10.1056/NEJMoa1612645 28564564

[12] von Minckwitz G, Huang CS, Mano MS, ; KATHERINE Investigators. Trastuzumab emtansine for residual invasive *HER2*-positive breast cancer. N Engl J Med. 2019;380(7):617-628. doi:10.1056/NEJMoa1814017 30516102

[13] Henke G, Knauer M, Ribi K, . Tailored axillary surgery with or without axillary lymph node dissection followed by radiotherapy in patients with clinically node-positive breast cancer (TAXIS): study protocol for a multicenter, randomized phase-III trial. Trials. 2018;19(1):667. doi:10.1186/s13063-018-3021-9 30514362 PMC6278139

[14] Weber WP, Matrai Z, Hayoz S, . Tailored axillary surgery in patients with clinically node-positive breast cancer: preplanned feasibility substudy of TAXIS (OPBC-03, SAKK 23/16, IBCSG 57-18, ABCSG-53, GBG 101). Breast. 2021;60:98-110. doi:10.1016/j.breast.2021.09.004 34555676 PMC8463904

[15] von Elm E, Altman DG, Egger M, Pocock SJ, Gøtzsche PC, Vandenbroucke JP; STROBE Initiative. The Strengthening the Reporting of Observational Studies in Epidemiology (STROBE) statement: guidelines for reporting observational studies. J Clin Epidemiol. 2008;61(4):344-349. doi:10.1016/j.jclinepi.2007.11.008 18313558

[16] Mittendorf EA, King TA, Tolaney SM. Impact of RxPONDER and monarchE on the surgical management of the axilla in patients with breast cancer. J Clin Oncol. 2022;40(29):3361-3364. doi:10.1200/JCO.22.00173 35675571

[17] Agostinetto E, Vian L, Caparica R, . CDK4/6 inhibitors as adjuvant treatment for hormone receptor-positive, *HER2*-negative early breast cancer: a systematic review and meta-analysis. ESMO Open. 2021;6(2):100091. doi:10.1016/j.esmoop.2021.100091 33743330 PMC8010395

[18] Appelgren M, Sackey H, Wengström Y, ; SENOMAC Trialists’ Group. Patient-reported outcomes 1 year after positive sentinel lymph node biopsy with or without axillary lymph node dissection in the randomized SENOMAC trial. Breast. 2022;63:16-23. doi:10.1016/j.breast.2022.02.013 35279508 PMC8920917

[19] Ashikaga T, Krag DN, Land SR, ; National Surgical Adjuvant Breast, Bowel Project. Morbidity results from the NSABP B-32 trial comparing sentinel lymph node dissection vs axillary dissection. J Surg Oncol. 2010;102(2):111-118. doi:10.1002/jso.21535 20648579 PMC3072246

[20] Fleissig A, Fallowfield LJ, Langridge CI, . Postoperative arm morbidity and quality of life. Results of the ALMANAC randomised trial comparing sentinel node biopsy with standard axillary treatment in the management of patients with early breast cancer. Breast Cancer Res Treat. 2006;95(3):279-293. doi:10.1007/s10549-005-9025-7 16163445

[21] Mansel RE, Fallowfield L, Kissin M, . Randomized multicenter trial of sentinel node biopsy vs standard axillary treatment in operable breast cancer: the ALMANAC Trial. J Natl Cancer Inst. 2006;98(9):599-609. doi:10.1093/jnci/djj158 16670385

[22] Giuliano AE, Hunt KK, Ballman KV, . Axillary dissection vs no axillary dissection in women with invasive breast cancer and sentinel node metastasis: a randomized clinical trial. JAMA. 2011;305(6):569-575. doi:10.1001/jama.2011.90 21304082 PMC5389857

[23] Giuliano AE, Ballman KV, McCall L, . Effect of axillary dissection vs no axillary dissection on 10-year overall survival among women with invasive breast cancer and sentinel node metastasis: the ACOSOG Z0011 (alliance) randomized clinical trial. JAMA. 2017;318(10):918-926. doi:10.1001/jama.2017.11470 28898379 PMC5672806

[24] Galimberti V, Cole BF, Viale G, ; International Breast Cancer Study Group Trial 23-01. Axillary dissection versus no axillary dissection in patients with breast cancer and sentinel-node micrometastases (IBCSG 23-01): 10-year follow-up of a randomised, controlled phase 3 trial. Lancet Oncol. 2018;19(10):1385-1393. doi:10.1016/S1470-2045(18)30380-2 30196031

[25] Bartels SAL, Donker M, Poncet C, . Radiotherapy or surgery of the axilla after a positive sentinel node in breast cancer: 10-year results of the randomized controlled EORTC 10981-22023 AMAROS trial. J Clin Oncol. 2023;41(12):2159-2165. 36383926 10.1200/JCO.22.01565

[26] Comparison of Axillary Lymph Node Dissection With Axillary Radiation for Patients With Node-Positive Breast Cancer Treated With Chemotherapy. ClinicalTrials.gov identifier: NCT01901094. Updated May 3, 2023. Accessed November 12, 2022. https://clinicaltrials.gov/ct2/show/NCT01901094

[27] Gerber B, Schneeweiss A, Möbus V, . Pathological response in the breast and axillary lymph nodes after neoadjuvant systemic treatment in patients with initially node-positive breast cancer correlates with disease-free survival: an exploratory analysis of the GeparOcto trial. Cancers (Basel). 2022;14(3):521. doi:10.3390/cancers14030521 35158789 PMC8833390

[28] Ryu JM, Choi HJ, Park EH, ; Korean Breast Cancer Society. Relationship between breast and axillary pathologic complete response according to clinical nodal stage: a nationwide study from Korean Breast Cancer Society. J Breast Cancer. 2022;25(2):94-105. doi:10.4048/jbc.2022.25.e17 35506578 PMC9065358

[29] Lim DW, Greene BD, Look Hong NJ. Relationship between breast and axillary pathologic complete response in women receiving neoadjuvant chemotherapy for breast cancer. Ann Surg Oncol. 2021;28(10):5495-5506. doi:10.1245/s10434-021-10519-8 34374914

[30] Del Mastro L, De Placido S, Bruzzi P, ; Gruppo Italiano Mammella (GIM) investigators. Fluorouracil and dose-dense chemotherapy in adjuvant treatment of patients with early-stage breast cancer: an open-label, 2 × 2 factorial, randomised phase 3 trial. Lancet. 2015;385(9980):1863-1872. doi:10.1016/S0140-6736(14)62048-1 25740286

[31] Moebus V, Jackisch C, Lueck HJ, . Intense dose-dense sequential chemotherapy with epirubicin, paclitaxel, and cyclophosphamide compared with conventionally scheduled chemotherapy in high-risk primary breast cancer: mature results of an AGO phase III study. J Clin Oncol. 2010;28(17):2874-2880. doi:10.1200/JCO.2009.24.7643 20458045

