# Supplementary material for: Association of Axillary Dissection With Systemic Therapy in Patients With Clinically Node-Positive Breast Cancer
Source: JAMA Surg. 2023 Jul 19;158(10):1013–21. doi: 10.1001/jamasurg.2023.2840 (PMC10357358; doi:10.1001/jamasurg.2023.2840)
Supplement: Supplement 1. — eTable. Logistic Regression Model for the Effect of Treatment Arm on the Administration of Adjuvant Systemic Therapy and Chemotherapy [file jamasurg-e232840-s001.pdf]

Supplementary Online Content

Weber WP, Matrai Z, Hayoz S, et al; TAXIS Study Writing Group. Association of axillary dissection with systemic therapy in patients with clinically node-positive breast cancer. *JAMA Surg*. Published online July 19, 2023. doi:10.1001/jamasurg.2023.2840

eTable. Logistic Regression Model for the Effect of Treatment Arm on the Administration of Adjuvant Systemic Therapy and Chemotherapy

This supplementary material has been provided by the authors to give readers additional information about their work.

© 2023 Weber WP et al. *JAMA Surgery*.

eTable. Logistic Regression Model for the Effect of Treatment Arm on the Administration of Adjuvant Systemic Therapy and Chemotherapy

	Unadjusted		Adjusted	
	**Odds ratio**	**95% CI**	**Odds ratio**	**95% CI**
**Upfront surgery setting: HR+HER2-**				
Adjuvant systemic therapy	0.67	(0.24 - 1.86)	0.50	(0.15 - 1.64)
Adjuvant chemotherapy	0.74	(0.22 - 2.46)	0.72	(0.19 - 2.67)
**Neoadjuvant chemotherapy**				
Adjuvant systemic therapy	0.92	(0.49 - 1.75)	0.86	(0.43 - 1.70)
Adjuvant chemotherapy	1.31	(0.85 - 2.00)	1.26	(0.78 - 2.01)

Multivariable analyses adjusted for palpable vs. non-palpable disease, menopausal status, tumor subtype, grade, age, year and country.

© 2023 Weber WP et al. *JAMA Surgery*.
