# Supplementary material for: Association of Axillary Dissection With Systemic Therapy in Patients With Clinically Node-Positive Breast Cancer
Source: JAMA Surg. 2023 Jul 19;158(10):1013–21. doi: 10.1001/jamasurg.2023.2840 (PMC10357358; doi:10.1001/jamasurg.2023.2840)
Supplement: Supplement 2. — Data Sharing Statement [file jamasurg-e232840-s002.pdf]

Data Sharing Statement

Weber. Association of Axillary Dissection With Systemic Therapy in Patients With Clinically Node-Positive Breast Cancer. *JAMA Surg*. Published July 19, 2023.
doi:10.1001/jamasurg.2023.2840

Data
Data available: Yes
Data types: Deidentified participant data
How to access data: Upon individual request to walter.weber@usb.ch.
When available: With publication

Supporting Documents
Document types: None

Additional Information
Who can access the data: Anyone with the respective approval(s).
Types of analyses: Any kind of analysis with the respective approval(s).
Mechanisms of data availability: After approval(s) and with a signed data transfer agreement.
